# A review of current knowledge about the importance of iodine among women of child-bearing age and healthcare professionals

**DOI:** 10.1017/jns.2022.50

**Published:** 2022-07-08

**Authors:** Lucy Kayes, Karen R. Mullan, Jayne V. Woodside

**Affiliations:** 1 Centre for Public Health, Queen's University Belfast, Institute of Clinical Sciences Building A, 294 Grosvenor Road, Belfast BT12 6BJ, UK; 2 Regional Centre for Endocrinology and Diabetes, Royal Victoria Hospital, 294 Grosvenor Road, Belfast BT12 6BA, UK

**Keywords:** Awareness, Iodine, Knowledge, Pregnancy

## Abstract

Iodine is required for thyroid hormone synthesis and fetal neurogenesis. Recent population studies in the United Kingdom (UK) have found iodine deficiency among schoolgirls, women of child-bearing age and pregnant women. This review explores knowledge and awareness of iodine among women of child-bearing age and healthcare professionals (HCPs) in the UK, set within a global context. We aimed to identify gaps in iodine knowledge in the current UK setting of iodine deficiency without iodine fortification and where iodine is not included in antenatal guidelines. The search terms ‘iodine knowledge’ and ‘iodine awareness’ were used to identify relevant papers. Iodine knowledge is poor among women of child-bearing age in the UK according to four studies using questionnaires and qualitative methods. They were unsure of dietary sources of iodine and were not consistently provided with relevant information from HCPs during clinical care. Midwives have been recognised as the main providers of dietary information during pregnancy and, although they recognised the importance of their role in providing nutrition advice, they did not feel equipped to do so and lacked confidence in addressing nutritional concerns. Globally, there was a similar lack of knowledge, however, this was somewhat improved by the inclusion of iodine in antenatal care guidelines. Midwives’ knowledge of iodine was poor, as was knowledge among women of child-bearing age. Improved HCP knowledge and effective communication of information to pregnant women and women planning to conceive may help to improve iodine status which is of particular concern in pregnancy.

## Introduction

Iodine is an essential component of thyroid hormones; required for normal cognitive function and metabolism throughout life^(1)^. During pregnancy, maternal iodine and thyroid hormones are required for fetal neurogenesis, including axon and dendrite growth, synapse formation, myelination and neuronal migration. Iodine is solely obtained from dietary sources, primarily dairy products, and seafood in the United Kingdom (UK), or supplements^(2)^. At the moment, in the UK, there is no requirement for iodine fortification and iodised salt is available in very few retail outlets^(3,4)^, while antenatal care guidelines from the National Institute for Health and Care Excellence (NICE) make no mention of iodine nutrition^(5)^. The most recent National Diet and Nutrition Survey found that, although children met the World Health Organization (WHO) definition of iodine sufficiency, women of child-bearing age (16–49 years of age) did not^(6)^.

Iodine requirements increase by approximately 50 % during pregnancy, leaving this population potentially vulnerable to iodine deficiency^(1)^. This may be compounded by the exclusion of iodine-rich foods due to the following of vegetarian or vegan dietary patterns or the exclusion of foods during pregnancy such as smoked fish, raw shellfish, marlin and shark as well as advising to limit portions of oily fish to two per week due to concerns regarding heavy metal toxins, as well as difficulties with the symptoms of nausea and vomiting^(7)^. Iodine deficiency in pregnancy is the leading cause of mental retardation in children worldwide^(8,9)^. The effects of severe iodine deficiency on fetal development are well recognised^(10,11)^; the effects of mild to moderate iodine deficiency are less well established^(11)^. It has been suggested that mild iodine deficiency in pregnancy may result in lower intelligence quotient (IQ) scores in offspring; as well as increased risk of perinatal complications^(12,13)^. Some population studies have supported this, as maternal iodine deficiency-induced thyroid dysfunction has been, albeit inconsistently, linked to impairments in intellectual and behavioural function in offspring^(1,14–16)^.

Historically, in the UK, there was a high prevalence of endemic goitre due to iodine deficiency; changes in livestock feeding patterns and government endorsement of cows’ milk intake improved the iodine status of the UK population and eradicated endemic goitre by the middle of the 20th century. Despite the need for regular population monitoring being noted, the iodine nutrition status of the UK population was not routinely monitored for many years^(17)^. Outside of the UK, iodine fortification in the form of iodised salt has been implemented in a number of countries since its recommendation by the WHO in 1993^(18)^. For example, in 1996, China implemented a universal salt iodisation policy following in the footsteps of the United States of America and Switzerland who have iodised salt since the 1920s^(19,20)^.

In 2009, Vanderpump studied the urinary iodine concentration (UIC) of schoolgirls (14–15 years old) across the UK and found this population to be iodine deficient. The prevalence of iodine deficiency was highest in the Belfast centre, with 85 % of participants having UIC within the deficient range as defined by the WHO (<100 μg/l)^(21)^.

A further cohort from the island of Ireland, which included a centre in Belfast, showed low-level sufficiency in the same schoolgirl age range^(22)^. A study of pregnant women from the same Belfast centre showed iodine deficiency, with a median UIC below the WHO recommended cut-off in pregnancy (150 μg/l), despite 55 % of participants reporting taking a supplement containing iodine^(23)^. This highlights the vulnerability of pregnant populations to iodine deficiency, particularly in the recently demonstrated precarious setting of low-level sufficiency as observed in the school-age population in the UK^(24)^. Improving awareness of this issue via public health campaigns targeting vulnerable groups may play an important role in improving iodine nutrition, along with consideration of fortification, supplementation during pregnancy and lactation, and midwife education^(25)^.

Pregnancy is often seen as an opportunity to effect behavioural change, as women are motivated and contact with health professionals is routine^(25–27)^. There is the unique motivation to have a healthy pregnancy, delivery and baby, but there are still barriers to positive lifestyle change including lack of knowledge^(27)^. Therefore, care received during pregnancy and the perinatal period may help to capitalise on a woman's motivation by helping them overcome the relevant barriers and providing advice on relevant nutritional issues^(26,27)^. However, there have been few studies exploring knowledge of iodine among women of child-bearing age and the healthcare professionals (HCPs) involved in providing antenatal care. Without this knowledge, behavioural change to improve iodine status is unlikely to occur^(26)^.

The Scientific Advisory Committee on Nutrition (SACN) reviewed the topic of iodine nutrition in 2014, calling for further research into iodine intake in the UK, particularly in vulnerable groups including during pregnancy and lactation, prior to recommendations regarding fortification or supplementation being made^(28)^. Meanwhile, despite the lack of high-quality evidence or trials concerning the effect of iodine supplementation on fetal outcomes, a number of health authorities worldwide, including in Australia and Croatia where iodine fortification is mandatory, have decided to include the recommendation for iodine supplementation during pregnancy and lactation in their guidelines for antenatal care^(29,30)^.

Despite the public health campaigns to improve awareness of and uptake of folic acid, another micronutrient with a vital role during early pregnancy, less than half of women are aware of the role of folic acid or the need to take supplements in the perinatal period^(31)^. This is further complicated by the rate of unplanned pregnancy in the UK of 45 %^(32)^. Pregnant women in the UK are entitled to a free ‘Bounty Pack’, which is often provided at their booking clinic appointment. This includes various information and advertising material some of which support the use of Vitabiotics Pregnacare, the leading pregnancy supplement in the UK. All varieties of Pregnacare contain iodine in the form of potassium iodide at a dose of between 140 and 200 μg/d^(33)^. However, there has been criticism of the timing of this as the first trimester, which is the key time for neurodevelopment, has already been completed and therefore any benefit may be limited.

This literature review aims to identify and evaluate studies relating to knowledge of the role of iodine during pregnancy among HCPs and patients in the UK. This will be set in the context of iodine knowledge globally. This will summarise the current evidence base, particularly while more data regarding iodine intake and deficiency in vulnerable populations, including during pregnancy, as requested by SACN, is generated to allow appropriate decision-making regarding the implementation of iodine supplementation during pregnancy and lactation and mandatory fortification of foods such as salt.

## Methods

To capture relevant papers, the term ‘iodine knowledge’ was entered into the ‘PubMed’ search engine and the Queen's University Belfast (QUB) library catalogue. Search filters to include papers available in English language were used. Searches were completed between October 2020 and April 2021. Women of child-bearing age were defined as women between 18 years and 45 years old. Professions included under the title of HCP were midwives, dieticians and doctors (of any speciality). Abstracts were reviewed for relevancy and those papers looking at iodine knowledge of HCPs and patients were downloaded in full and separated into the UK and international papers. Papers were then reviewed in full by one author (LK); initially only those that specifically mentioned iodine knowledge were included, but as there were so few papers relating to HCPs knowledge of iodine, papers that had been found during the search with a more general focus on nutrition knowledge of HCPs in pregnancy were also considered eligible. The search was then repeated using the term ‘iodine awareness’ and similar process followed.

### Patient knowledge

Four papers examining iodine knowledge of patients within the UK were identified. Most studies used questionnaires while one used qualitative methodology. Table 1 shows a summary of the findings from studies of patient iodine knowledge in the UK. Key themes from studies in the UK were that iodine knowledge is low among women of child-bearing age, they are unsure of dietary sources of iodine and they are not consistently provided with relevant information from HCPs^(34–37)^.

**Table 1.** Summary of papers from the UK reporting iodine knowledge in women of child-bearing age

Author (Publication Year)	Participants (*n*)	Method	General Themes/Results	Iodine Specific Themes/Results
Bouga *et al*. (2018)^(2^^)^	Pregnant, breast-feeding, planning to start a family or trying to conceive or child <2 years old (48)	Interviews and thematic analysis	100 % had heard of folic acid and 75 % had heard of vitamin D. 22 % reporting cutting out a type of seafood or fish while pregnant. 40 % reported increasing dairy intake during pregnancy.	23 % had heard about iodine. 25 % aware of the role of iodine in fetal development. 72 % of participants had low confidence achieving adequate iodine intake.
Combet *et al.*(2015)^(35)^	Pregnant women (1026)	Questionnaire	96 % reported awareness of dietary and lifestyle recommendations specific for pregnancy. 59 and 52 % reported written and oral advice from doctor, respectively, as source of information. 47 % reported confusion around the recommendations. 82 % used the internet for complementary information. 18 % got complementary information from HCPs. 64 % had never received information about iodine and only 11 % had heard about iodine from HCPs.	12 % reported information on iodine was sufficient. 56 % were unable to identify any iodine-rich food. 9 % recognised milk and 6 % yoghurt as iodine-rich sources. 84 % were unaware the iodine is important for healthy fetal development during pregnancy. 85 % agreed or strongly agreed that they would increase their iodine intake if they were aware of the impact of iodine deficiency.
McMullan *et al.* (2019)^(36^^)^	Pregnant women (183)	Questionnaire	N/A	9 % identified dairy products as a good source of iodine. 20 % answered that iodine requirements increased during pregnancy and breast-feeding. 5 % identified that iodine deficiency could be harmful. 5 % of participants agreed/strongly agreed that adequate information was provided on iodine.
O'Kane *et al*. (2016)^(37)^	Women of child-bearing age (520)	Questionnaire	N/A	9 % had previously received information about iodine (mostly during formal education) and this subgroup had a significantly greater general awareness. 43 % were aware of the nutrient iodine. 41 % did not know or could not identify any health problem associated with iodine deficiency. 9 % identified milk and dairy products as a rich source of iodine. 27 % were aware the iodine deficiency is a current public health concern in the UK.

There was general agreement among participants that there was a lack of information provided on iodine. In the cohort studied by Bouga *et al.*, a quarter of women reported no discussion around nutrition in pregnancy and that advice regarding iodine was insufficient, with education, when given, focusing on foods to avoid and dietary supplements^(38)^. In the cohort of women of child-bearing age studied by O'Kane *et al.*, only 9 % had previously received information about iodine and 75 % reported this was during formal education rather than as part of clinical care^(37)^. The 12 % of participants in the study by Combet *et al.* who reported receiving sufficient iodine information in pregnancy did not have a significantly higher iodine intake, however, the 64 % who had received sufficient information on calcium did have significantly higher iodine intakes from food and supplements throughout pregnancy compared with those who had reported not receiving sufficient information^(35)^. It was also noted that multiparous women reported receiving less information in their current pregnancy than during their first pregnancy, as prior knowledge was assumed^(36,38)^.

Women were unaware of dietary sources of iodine. In the study by Combet *et al.*, 56 % were unable to identify any iodine-rich food, but incorrectly identified dark green vegetables and salt as good sources of iodine in the UK, where salt is not required to be fortified with iodine and there is low availability of iodised salt^(3,4,35)^. This was similar to the 45 % of participants who were unsure of dietary sources of iodine in the cohort studied by McMullan *et al.*^(36)^. Milk and dairy products, the largest dietary contributors to iodine intake in the UK, were identified as iodine rich by only 9 % of women in this study and a similar proportion of the participants in the study by O'Kane *et al.*^(36,37)^. A great percentage of women in both cohorts identified fish as an iodine-rich food source with 30 and 39 %, respectively^(36,37)^.

The study by O'Kane *et al.* was advertised through a local higher education institution, resulting in a cohort with greater knowledge than would be expected in the general population and included women of child-bearing age rather than those who are or have been pregnant, and therefore participants may not have been exposed to antenatal care. Despite this assumed greater knowledge, 46 % of participants did not meet the recommended daily intake of iodine calculated by food frequency questionnaire and few correctly identified dietary sources of iodine^(37)^. This and one other study reported that increased maternal age and higher educational attainment were associated with significantly higher total iodine knowledge scores^(35,37)^. Higher intake of iodine-rich foods (fish and dairy products) was positively associated with iodine knowledge and knowledge of health problems associated with iodine deficiency^(2,34,36,37)^.

There was, in general, a willingness to change dietary behaviours in pregnancy when provided with specific advice by HCPs with supplementary information for future reference^(35)^. For example, women reported reducing or eliminating fish in their diets due to concern regarding heavy metals and toxins resulting in potential harm to their baby, following nutrition advice^(35,38)^.

### Professional knowledge

Four papers relating to UK HCPs’ iodine and nutrition knowledge were reviewed. Table 2 shows a summary of these papers. In the UK, midwives provide routine antenatal care and community midwives have been recognised as the main providers of dietary information during pregnancy^(2)^. The papers highlight that, although midwives are aware of the importance of their role as nutrition advisors, they do not feel adequately equipped to perform it and lack confidence in addressing nutritional concerns^(2,39–42)^.

**Table 2.** Summary of papers discussing midwife knowledge of nutrition and iodine in the UK

Author (Publication Year)	Participants (*n*)	Method	Themes/Results
Mulliner *et al.* (1995)^(39)^	Midwives (58)	Questionnaire followed by semi-structured interview	24 % reported their training as the most influential factor in their confidence in discussing diet. 48 % stated the media was a key source of confidence for discussing nutritional issues. 53 % had gained most of their information from the media. 66 % concerned about their level of knowledge when discussing vegetarian diet. 93 % saw a need for a set of nutritional guidelines.
Barrowclough and Ford (2001)^(40)^	Midwives (27)	Questionnaires	Significant increase in knowledge scores following completion of nutrition open-learning material. Only five midwives were aware of the recommendations for the daily increase in calories in the last trimester of pregnancy. Midwives had poor knowledge of how to identify women at risk of iron-deficiency anaemia. There was confusion around folic acid supplementation, particularly around the appropriate dose to prevent recurrence of a neural tube defect. No specific information about iodine.
Williamson *et al.* (2012)^(41)^	Midwives (60)	Questionnaires administered face-to-face	95 % identified the need for further education. 20 % could link iodine deficiency with impaired fetal neural development. 10 % were aware of increased iodine requirements in pregnancy. 23 % identified milk/dairy as iodine-rich foods. All respondents were ‘unconfident’ regarding all aspects of iodine in pregnancy.
McCann *et al.* (2017)^(42)^	Midwives (17)	Semi-structured interviews	Midwives lack resources and expertise. Midwives normalised obesity. All midwives agreed with the NICE recommendation that midwives should provide healthy eating advice. Practical limitations to current clinical practice. No specific information about iodine.

The study by McCann *et al.* focused on obesity and discussed midwives’ experiences of weight management advice as well as the broader topic of healthy eating. This cohort reported high workload, along with the normalisation of obesity, and lack of knowledge as reasons for the lack of dietary advice provided as part of routine antenatal care. The midwives in this study were aware of the health risks associated with poor diet and obesity in pregnancy but lacked the confidence and skills to give appropriate advice^(42)^. It was recognised that a lack of support in midwifery training may impact on the qualified midwife's ability to perform their role as nutrition educator, and many midwives indicated the need for further education^(39,42)^.

Barrowclough *et al.* developed open-learning materials for midwives on the topic of perinatal nutrition. Participants had a statistically significant increase in knowledge score after completion of training via the materials. Topics included weight gain and recommended an increase in calorie intake during pregnancy, folic acid supplementation and iron-deficiency anaemia^(40)^. However, it was noted that this style of learning is time-consuming, with a minimum of 6 h required to complete this package determined during a pilot study, although the time taken by each participant was not recorded. Implementation of this style of learning would require support from managers to ensure sufficient study time is allowed for busy practicing midwives^(40)^. This open-learning pack did not include the topic of iodine nutrition, although it could be developed to include this and other topics at a variety of academic levels^(40,43)^.

The papers by Mulliner *et al.* and Williamson *et al.* addressed the subject of iodine knowledge in HCPs, but only included midwives. There are similar findings in the two studies despite 17 years having elapsed between their publication. Each study encompassed only one health authority within the UK, and both included small numbers (58 and 60, respectively) when compared with the estimated 39 000 currently registered midwives within the UK^(44)^. In both cohorts, a need for further education and clinical guidelines was reported^(39,41)^.

The questionnaire used by Mulliner *et al.* focused on knowledge and a marking scheme was established by circulating the relevant section to a dietician, a consultant obstetrician and three midwives and comparing key areas identified by them with the researcher's expectation. They report that consensus was reached without difficulty and key questions were given extra weight^(39)^. In contrast, Williamson *et al.* asked midwives to rate their confidence on a Likert scale. Most midwives in this cohort saw women in the last two trimesters of pregnancy, 18 % were community based and only 2 % routinely provided advice on iodine nutrition^(41)^. Participants rated knowledge on general nutrition ‘very confident’ and ‘confident’ for dietary guidelines, but ‘unconfident’ for all aspects of iodine nutrition, without any formal assessment of knowledge^(41)^.

Since the publication of Mulliner *et al.* in 1995, NICE have published guidance on maternal and child nutrition, and antenatal care. However, neither guideline includes any information around iodine nutrition in pregnancy, instead focusing on folic acid and foods to be avoided in pregnancy^(5)^. The omission of iodine from the NICE guidelines and other antenatal resources, such as the ‘Pregnancy Book’ in Northern Ireland and ‘Ready, steady, baby!’ in Scotland, may reflect the lack of iodine awareness among professionals and policymakers, as well as the ongoing need for good quality evidence as highlighted by SACN^(45)^.

Midwives reported lack of time, lack of knowledge and lack of confidence as the barriers to providing nutrition education in pregnancy^(39)^. The importance of nutrition as part of health promotion periconceptually has been recognised, but, given the large amount of knowledge midwives have to gain during their undergraduate studies, it seems to be less of a priority during this time^(39)^.

There is currently no information regarding UK clinicians’, and other HCPs’, knowledge of iodine nutrition and, although the General Medical Council recommend that doctors should be able to meet support patients to meet their nutritional needs, a study of final year medical students found they had poor knowledge of nutrition^(46)^. This means the lack of knowledge among midwives may not be offset by other HCPs.

## International context

Globally, as in the UK, there is a lack of iodine knowledge among women and HCPs. Five papers from outside the UK focusing on patient iodine knowledge were examined. Table 3 shows a summary of findings from studies around the globe regarding patients’ iodine knowledge. Two of these were from Australia where there has been a focus on iodine nutrition recently following the re-emergence of iodine deficiency and the introduction of iodine fortification. Iodine knowledge has improved post-fortification and more women reported receiving adequate information on iodine during pregnancy after the publication of the updated National Health and Medical Research Council (NHMRC) guidelines including iodine^(47)^.

**Table 3.** Summary of international papers discussing women of child-bearing age's iodine knowledge.

Author (Publication Year)	Country	Participants (*n*)	Method	Themes/Results	Iodine status (% deficient)
Charlton *et al.* (2012)^(47)^	Australia	Pregnant (139) and non-pregnant (75) women in 2007–08. Pregnant (147) and lactating (60) women in 2011–12.	Questionnaire	64 % were unsure if their diet provided adequate iodine for their needs. 5 % identified bread as legally required to have iodine added. 25–33 % identified milk, eggs or bread as a good source of iodine. 50–58 % identified seafood as a good source of iodine. 27–31 % identified mental retardation as a potential outcome of iodine deficiency. 83 and 66 % pre- and post-fortification, respectively, felt they had not received enough information regarding iodine during pregnancy to make informed decisions. 37 % received verbal information regarding iodine from HCP while 12 % received written information on the subject. 11 and 22 % pre- and post-fortification, respectively, reported changing their diet once pregnant to increase iodine intake.	71⋅5 %
Martin (2014)^(64)^	Australia	Pregnant women (200)	Questionnaire	62 % reported taking an iodine containing supplement, however 8 % contained less than the recommended 150 μg of iodine. 3 % ate fish 2–3 times per week. 19 % indicated limiting or avoiding seafood due to listeriosis or mercury contamination. 19⋅5 % reported consuming iodised table salt daily. 18⋅5 % indicated the need for an iodine supplement in pregnancy. 15⋅5 % of women were aware of the iodine fortification programme. 54⋅5 % had heard about the importance of increasing iodine in pregnancy. 83⋅5 % of women reported medical practitioners as their main source of information for pregnancy and health. 34⋅3 % selected medical practitioners as advisors on the importance of iodine.	71⋅5 %
Garnweidner-Holme (2017)^(65)^	Norway	Pregnant (804) and lactating (175) women	Questionnaire	74 % of pregnant and 55 % of lactating women achieved a score of none or low iodine knowledge. 16⋅6 % of pregnant and 7⋅4 % of lactating women had received information on iodine from HCPs. 5 % of pregnant women reported they were confident they could achieve the recommended daily intake of iodine through their diet. 37⋅4 % of women did not know the important dietary sources of iodine. 70 % were unsure if iodine intake was a current public health concern in Norway.	No data
Vidranski *et al.* (2019)^(30)^	Croatia	Women of reproductive age (378)	Questionnaire	53 % were not familiar with the role of iodine in the body. 62 % did not know that salt was iodised by law in Croatia. 20 % reported thyroid disease.	28⋅8 %
Wang *et al.* (2020) ^(20)^	China	Pregnant women (2400)	Questionnaire followed by interview	71⋅5 % of households used iodised salt (average iodine content 23⋅9 mg/kg). 30⋅3 % lack iodine-related knowledge. 62⋅8 % of pregnant women have a habit of consuming iodine-rich food.	16⋅2 %

HCP, healthcare professional; N/A, not applicable.

Iodine status is taken as the proportion of population with urinary iodine concentration <100 μg/l (deficient) from the most recent World Health Organization survey, data collected 1993–2003.

As in the UK, midwives are the main providers of antenatal care and their role as a source of health and nutrition information in pregnancy has been recognised in Australia^(48)^. However, their ability to provide the necessary information may be limited by a lack of knowledge and skills. The midwifery undergraduate curriculum includes nutrition, although teaching hours are reported to be low, and the focus not on practical training^(49)^. In one survey, most midwives had received nutrition education, but it was generally described as limited. The cohort in this study, as in others, reported a need for further nutrition education, with 94⋅2 % feeling this would benefit their clinical practice^(39,49)^.

Awareness of the updated NHMRC guidelines was a statistically significant predictor of advising on iodine supplementation during pregnancy^(50)^. Midwives were the least likely HCPs to recommend iodine supplements, with dietitians being the most likely followed by general practitioners, and obstetricians and gynaecologists. Most midwives reported a lack of knowledge as the main reason for not discussing dietary sources of iodine. This was different from other HCPs, who stated lack of time as the main reason for not discussing this topic. In the present study, 57 % of midwives were aware of the NHMRC recommendation for iodine supplementation, although only 18 % knew the recommended dose. Almost all HCPs, from all disciplines, were interested in receiving more information about iodine nutrition, in particular the NHMRC recommendations and dietary sources of iodine^(50)^.

The population studied by Wang *et al.* in China, a country with a well-established salt iodisation programme, showed a significant difference between the UICs of those with ‘poor iodine knowledge score’ and ‘high iodine knowledge score’. They found that iodine-related knowledge, having a habit of consuming iodine-rich food (including iodised salt), actively seeking dietary knowledge and trimester of pregnancy were associated with significantly higher UIC^(20)^.

Poor iodine knowledge has been recognised as a factor contributing to iodine deficiency^(37,47)^. However, improving iodine knowledge alone may not be sufficient for women to achieve iodine sufficiency in pregnancy, although it may affect behaviour around supplementation^(51,52)^ and these concerns are supported by the ongoing difficulties faced implementing changes to behaviour around folic acid for the prevention of neural tube defects (NTDs).

### Iodine in the context of folic acid

There are potential lessons to be learned from the delays in policymaking around folic acid, preconception supplementation and fortification in the UK. Despite the recommendations regarding folic acid that women take a 400 μg supplement of folic acid before pregnancy and for 12 weeks after conception^(5)^, and associated public health campaigns, compliance remains low^(53)^ with one study reporting that 31 % of women reported taking folic acid supplements as recommended by the NICE guidelines^(54)^. This low uptake is likely to be influenced by the rate of unplanned pregnancies and the fact that in the UK HCP contact and nutrition counselling occurs at the end of the first trimester.

In 2006, the SACN recommended fortification of flour with folic acid in the UK. This was due to the poor compliance with folic acid supplementation, particularly in deprived socio-economic groups, and the number of unplanned pregnancies in the UK making preconceptual supplementation difficult^(32,55)^. It has been estimated that 2000 births have been affected by NTDs between 1998 and 2012, that may have been prevented if folic acid fortification had been implemented in 1998, the same year it was made mandatory in the US^(56)^.

In one cohort, women reported receiving information on folic acid around 12 weeks gestation, at which time it was deemed redundant. However, the proportion of women taking folic acid does increase after confirmation of pregnancy^(57,58)^. Women also reported no particular emphasis on the topic during antenatal care^(54)^. This may be explained, as with iodine, by a lack of knowledge among HCPs as there was confusion around the recommendations for folic acid in a cohort of midwives in the UK^(40)^.

Factors linked to lower rates of folic acid supplement use are unintended pregnancy, age, socio-economic and ethnic group^(53)^. Initiatives, integrated into healthcare, can be effective, but are more likely to be successful if they include making supplements easily available. Using printed resources is less effective for women in lower socio-economic groups. There is also lower awareness of folic acid recommendations in these groups, and the current folic acid policy may exacerbate health inequalities^(53,57)^. In contrast, information received from HCPs has been associated with changes in modifiable behaviours, including taking folic acid supplements^(58–61)^.

## Future research

There is a need to review nutrition education within the current undergraduate curriculum for HCPs; including potentially, not only the transmission of knowledge, but practical instruction on motivational interviewing techniques to provide HCPs with the knowledge and confidence to discuss these issues with women prior to pregnancy and during antenatal care. This should overcome some of the barriers to providing nutrition information cited by midwives in the limited research currently available. Other barriers, including lack of time and the belief that discussion will not result in behavioural change, may be more difficult to tackle. Awareness of preconception health among women and HCPs is low and responsibility for providing preconception care is not well defined. This is problematic as first contact with HCPs is generally around 12 weeks gestation, after the initial period of fetal development. Modifiable factors, such as maternal diet and nutritional status, have an important influence on the intrauterine environment and fetal development^(61)^.

To examine the potential cost of implementation of iodine supplementation within the National Health Service, one group performed cost analysis of iodine supplementation for 13 weeks preconception continuing through pregnancy and lactation^(62)^. The model used was based on a conservative approach limiting the potential benefits of iodine supplementation and overestimating its potential harms. The main effect examined was of reduced IQ in offspring based on the economic and societal cost implication. A reduced IQ is associated with an increased rate of mortality with a higher risk of suicide and mental ill health as well as a higher incidence of cardiovascular disease. Overall, with a conservative estimate of a discounted lifetime value of an additional IQ point of £3297, iodine supplementation was cost-effective with a net gain of 1⋅22 IQ points^(62)^. Estimates of cost were based on earnings from the United States in 1974 and 1990 and do not take into account the advances in technology in the work place which may increase the value of an additional IQ point to today's workforce^(62)^.

The impact of iodine supplementation on maternal health is less clear. One meta-analysis showed no benefit of iodine supplementation in pregnant women with mild iodine deficiency; although this may be due to the physiological adaptation of the thyroid gland to maintain thyroid hormones at a normal level for fetal development, even in the setting of low iodine intake^(63)^. There were only five papers relating to maternal health outcomes. Differing formulations and doses of iodine supplementation were used and supplementation was commenced between 10 and 18 weeks gestation which may be too late to see benefit^(63)^.

The SACN report on iodine highlights the need for further research into the safety and efficacy of iodine supplementation in pregnancy, as well as the development of a biomarker of iodine deficiency at an individual level. There are practical and ethical issues limiting the study of iodine supplementation before preconception and during pregnancy and lactation. A placebo-controlled trial could be considered unethical if there is potential benefit from iodine supplementation as well as the practical difficulties of recruiting women early in pregnancy when fetal neurodevelopment takes place. However, the requirement for high-quality evidence to inform UK policy and guidelines is clear, the need for which were highlighted by practicing midwives^(39)^.

Our group is currently carrying out qualitative research aiming to understand UK midwives’ current iodine knowledge, their experience of nutrition education during their undergraduate studies and their views on their role and responsibility to provide nutrition information to women. We are also performing an intervention study with pregnant women completing an iodine knowledge questionnaire before and after the provision of an information leaflet as part of a larger study.

## Conclusion

Midwives’ knowledge of iodine and its role in healthy pregnancies is poor according to the limited studies to date within the UK. This is reflected by poor knowledge in women of child-bearing age, even in those who have received dietary advice as part of routine antenatal care. Globally, there was poor knowledge of iodine among HCPs, however the effects of this may be mitigated by the implementation of fortification and supplementation, such as in Australia and Croatia. This review is limited by the lack of a validated iodine knowledge questionnaire, making comparisons more difficult, and by the lack of data on iodine status on the UK within the latest WHO worldwide survey. Improving iodine status, particularly in pregnancy, should be a priority within the UK to prevent a similar missed opportunity as with folic acid. Improved HCP knowledge and effective communication of information to pregnant women or women planning to conceive is one step in this process. Australia may offer an example for the UK to follow, with several similarities, including poor HCP knowledge of iodine, although Australia has moved forward with the implementation of iodine supplementation in their latest antenatal guidelines. In the absence of mandatory iodine fortification, low availability of iodised salt, lack of guidance on iodine supplementation prior to or during pregnancy and lactation in the UK, raising HCP knowledge and awareness of iodine deficiency as a current public health concern in the UK is essential.
